# Pathophysiological Role of Global Cerebral Ischemia following Subarachnoid Hemorrhage: The Current Experimental Evidence

**DOI:** 10.1155/2013/651958

**Published:** 2013-06-12

**Authors:** Nikolaus Plesnila

**Affiliations:** Institute for Stroke and Dementia Research (ISD), University of Munich Medical Center, Ludwig-Maximilians-University, Max-Lebsche Platz 3, 81377 Munich, Germany

## Abstract

Subarachnoid hemorrhage (SAH) is the subtype of stroke with one of the highest mortality rates and the least well-understood pathophysiologies. One of the very early events which may occur after SAH is a significant decrease of cerebral perfusion pressure (CPP) caused by the excessive increase of intracranial pressure during the initial bleeding. A severely decreased CPP results in global cerebral ischemia, an event also occurring after cardiac arrest. The aim of the current paper is to review the pathophysiological events occurring in experimental models of SAH and global cerebral ischemia and to evaluate the contribution and the importance of global cerebral ischemia for the pathophysiology of SAH.

## 1. Introduction

Subarachnoid hemorrhage (SAH) is a relatively rare subtype of stroke (incidence: 10/100,000 person years; 5% of all first-ever strokes) which is characterized by the presence of blood in the subarachnoid space, the cerebrospinal fluid-filled space between the pia arachnoidea, a thin membrane which covers the brain parenchyma, and the dura mater [1–4]. The vast majority of SAHs (85%) is caused by the spontaneous rupture of a cerebral aneurysm located at the skull base. The consequence of blood being released into the subarachnoid space with a pressure almost equal to systolic blood pressure is that 20%–25% of patients die almost immediately after SAH [1]. From those patients reaching a hospital, 33% die within the first 30 days after hemorrhage and about 33% survive only with persisting neurological deficits making them dependent on daily care [2, 5]. The remaining 33% of patients were independent 18 months after SAH; however, only 1/3 of these patients reported no reduction in quality of life as compared to the premorbid state [6]. Accordingly, about 50% of SAHs are lethal and less than 8% of patients fully recover. Therefore, SAH is regarded as the subtype of stroke with the worst prognosis; due to the relatively young age at which SAH occurs, the loss of potential life before the age of 65 is comparable to that of ischemic stroke, a condition which is more than 20 times more frequent (incidence: 240/100,000 person years) [7].

Despite large technical and procedural achievements in the diagnosis of SAH, in the prevention of rebleedings, and in general intensive care over the past three decades, it is a matter of debate whether the outcome after SAH improved significantly [3, 5, 8, 9]. This disappointing situation may be the mere reflection of the severity of the disease, however, since most sequelae of SAH occur with a delay of several hours and even days, it is generally accepted that a deeper understanding of the pathophysiology of the secondary insults caused by the initial hemorrhage may be the key for the development of novel therapeutic strategies and, hence, for improving patient outcome. 

The CNS-related pathophysiological events following SAH can be divided into an early component and a delayed component. The delayed component occurs later than four days after hemorrhage and is characterized by delayed spasms of large intracranial vessels and possibly cerebral microvessels leading to cerebral ischemia in distinct areas of the brain, that is, focal cerebral ischemia. Delayed large artery spam has been studied extensively over the past 20 years, and endothelin 1 receptors were found to be the molecular surrogate for posthemorrhagic vasospasm [10]. Unfortunately, recent clinical evidence suggests that although endothelin receptor antagonists were able to reduce posthemorrhagic vasospasm, patient's outcome did not improve significantly [11]. Consequently, in recent years, research started to focus more on the early component of the pathophysiology of SAH [8, 12]. Another reason for this change of focus is certainly also that mortality during the first few days after SAH is four times higher than that during the late phase [1, 13, 14]. 

One of the main characteristics of early brain injury (EBI) following SAH is a severe reduction in cerebral blood flow in various regions of the brain [15] which may cause cortical spreading depolarization (CSD), spreading ischemia, and subsequent ischemic brain damage [16]. Interestingly, cerebral ischemia occurs under conditions of normal or almost normal cerebral perfusion pressure (CPP) suggesting that ischemia is caused by constriction of intracerebral vessels. Since neither clinical nor experimental evidence suggest that functionally relevant macrovasospasm is present at this early stage after SAH, the cerebral perfusion deficit has to be located on the level of the cerebral microcirculation. Indeed Uhl and colleagues and later Pennings and colleagues demonstrated already ten years ago that pial microvessels show pearl-string-like constrictions in SAH patients [17, 18]. Such microvasospasms were later also found in experimental studies using histological techniques [19] and *in vivo* imaging [20, 21]. 

Despite these interesting and clinically relevant findings explaining the occurrence of focal ischemic brain damage in the cortex and in the basal ganglia and the subsequent functional deficits observed after SAH, some other important features of the pathophysiology of SAH are still unclear. It is, for example, still unclear why SAH patients suffer from global brain edema [22, 23], why glutamate levels increase after SAH but decrease shortly thereafter [24], why in animal models of SAH neuronal injury is mainly observed in the hippocampus and not in the cerebral cortex [25], and why patients surviving SAH suffer from pronounced memory deficits [26]. These changes occur on top of early cortical ischemia and may be associated with global ischemia due to the exceedingly high increase in intracranial pressure and the resulting cessation of cerebral perfusion during and shortly after the initial vessel rupture as suggested by various authors already decades ago [27, 28]. Since the pathophysiological changes observed immediately, that is, within the first 60 minutes after SAH, cannot be investigated in patients, the aim of the current paper is to review the experimental literature and evaluate whether there is enough evidence to suggest that global cerebral ischemia is an important feature of the pathophysiology of SAH.

## 2. Pathophysiological Findings following Experimental SAH

A plethora of techniques and species were used during the past decades to study SAH under experimental conditions [29–32]. At the time when the emphasis of SAH research was mainly on delayed cerebral vasospasm, animal models able to reproduce this condition experimentally were predominantly developed [29–32]. The model of SAH and vasospasm most frequently used was the canine “two-hemorrhage” model, in which two injections of blood into the basal cistern were performed 48 hours apart. On the basis of its ability to accurately predict what occurs in human SAH, a primate model in which a blood clot is surgically placed around the large cerebral vessels at the base of the brain was used in dedicated centers [29]. After more recently scientists became also interested in early brain injury after SAH, animal models reproducing the early pathophysiology of SAH became more popular and more frequently used [30–32]. Among those models, the intravascular perforation model, where the Circle of Willis is perforated without craniotomy by an endovascular approach, seems to be the procedure which reproduces the early pathophysiology of SAH most adequately [30–33]. Therefore, most of the data currently reviewed derive from experiments performed with the filament perforation model.

**Figure 1:**
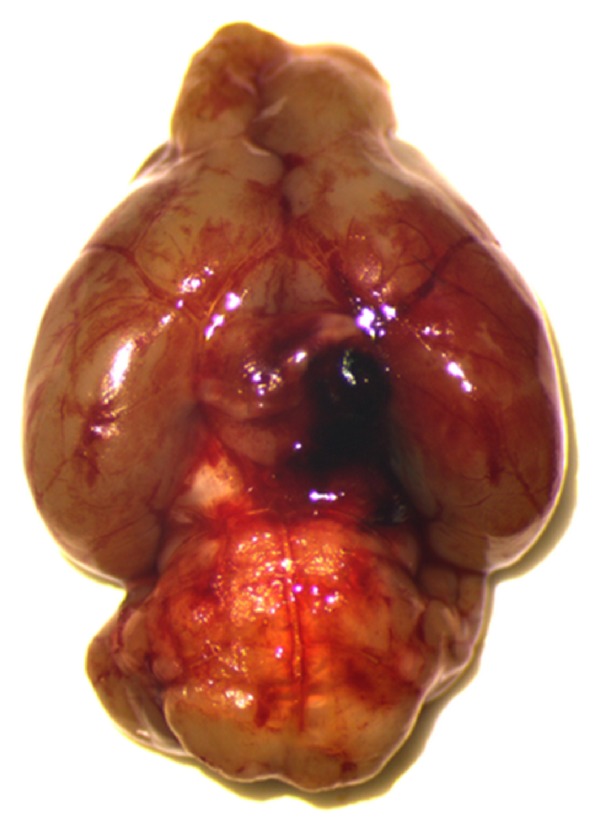
Perfused mouse brain three hours after experimental SAH (endovascular perforation model). A large clot formed at the perforation site (dotted white circle), and blood is distributed from the bleeding site into the subarachnoid space, preferentially along blood vessels.

**Figure 2:**
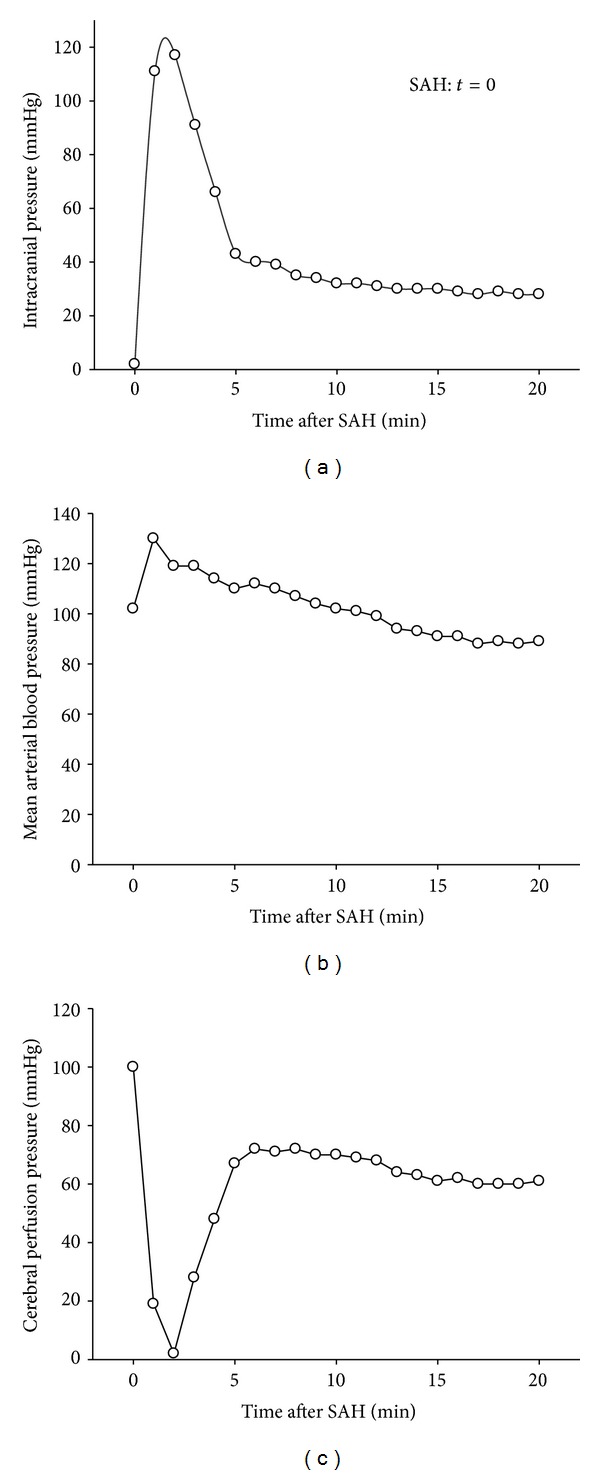
Intracranial pressure (ICP; (a)), mean arterial blood pressure (MAP; (b)) and cerebral perfusion pressure (CPP; (c)) after SAH in a mouse (*t* = 0; endovascular perforation model).

When inducing SAH experimentally by endovascular perforation of the Circle of Willis, blood is released into the subarachnoid space at the skull base where it forms a large clot (Figure 1). Since the growing clot uses up a significant proportion of the intracranial volume, the intracranial pressure (ICP) starts to rise immediately after the hemorrhage to values around 100 mmHg (Figure 2(a)). The immediate increase in ICP triggers an increase of blood pressure, the so-called Cushing Reflex (Figure 2(b)), thereby aggravating the bleeding [34]. The intracranial hypertension results in a pathological decrease of cerebral perfusion pressure (CPP) for up to 5 minutes (Figure 2(c)). This CPP decrease results in a global suspension of cerebral blood flow for 2-3 minutes [24, 33, 35–37] which is equal to global cerebral ischemia.

The stop of cerebral circulation together with local vasoconstriction and activation of the coagulation cascade promote the formation of a blood clot at the bleeding site and, hence, cessation of hemorrhage as indicated by a gradual decrease of ICP over the next 2-3 minutes to values around 30 mmHg. Consequently, CPP recovers to near normal values of 60 mmHg or more (Figure 2). Interestingly, many groups report that despite the recovery of CPP, CBF does not necessarily recover and may stay at low levels in both hemispheres for up to 60 min after SAH [24, 33, 35, 36]. Acute vasoconstriction of large intracerebral arteries was made responsible for this phenomenon [33]; however, this early lack of CBF recovery after SAH is prevented when instead of anesthetics with a known blood pressure-lowering and Cushing Reflex-suppressive effect, that is, halothane or isoflurane, anesthetics are used which maintain systemic blood pressure [38]. Hence, it remains unclear whether the prolonged CPP-independent drop of CBF after SAH is a pure experimental phenomenon or indeed a component of the early pathophysiology of SAH. 

No matter if CBF fully recovers or not, SAH results also in metabolic changes in the brain parenchyma as demonstrated by *in vivo* microdialysis [24, 33]. Glutamate increases up to sixfold already 30 min after SAH and gradually returns to near baseline values within the next 1.5 hours. This increase in glutamate is paralleled by an increase in the lactate/pyruvate ratio, an indicator of tissue ischemia [24]. Since microdialysis reflects the situation in the brain parenchyma only with a delay of up to 30 min (depending on sampling conditions), it is conceivable to conclude that the metabolic changes observed after SAH by microdialysis occur mainly immediately after the initial hemorrhage and are therefore a strong indicator for global cerebral ischemia [24]. 

Concomitant with the recovery of ICP, CPP, CBF, and tissue metabolism, the posthemorrhagic brain starts to display a slow but steady increase in brain water content from three to six hours until at least three days after SAH [25, 39, 40]. The delayed and slow development of brain edema suggests that the underlying pathophysiology may be linked to opening of the blood brain barrier (BBB) rather than to the initial posthemorrhagic global ischemia, since brain edema formation following global ischemia is caused by ischemic cell swelling and therefore disappears within minutes after reperfusion [41]. Indeed, injection of blood into the subarachnoid space of rats—a model devoid of all acute changes in ICP, CPP, and CBF described earlier—resulted in an increased vascular permeability brought about by focal disruption of endothelial tight junctions and the subsequent opening of the BBB in the underlying cortex [42]. The molecular mechanisms responsible for this BBB opening have not been fully elucidated but involve activation of matrix metalloproteinase 9 and degradation of the microvascular basal lamina [37, 43]. If one takes into consideration that in SAH patients and in animals subjected to SAH by endovascular puncture blood is distributed in the whole subarachnoid space, it is conceivable that under these conditions vasogenic brain edema will develop in all cortical regions of the brain and may therefore make a “global” impression. When extrapolating to the human situation, it is, hence, very likely that the global edema observed in patients [22, 23, 44] is just the reflection of blood-induced microvascular leakage and has little or no pathophysiological link to the global ischemia observed immediately after the initial bleeding.

SAH in mice and rats is accompanied by a mortality of 35%–50% mainly between 24 and 72 hours after vessel perforation [25, 29, 30, 33, 37, 45], values well comparable to those observed in SAH patients [1, 2]. At least in experimental animals, the reason for this mortality is certainly not related to focal brain ischemia due to delayed vasospasm since rodents do not develop symptomatic large artery spasms neither at the time when mortality occurs nor later [31]. Accordingly, rebleedings or the sequels of early brain injury (EBI) have to be involved; however, the underlying mechanisms are by far not fully understood yet [8, 12, 46]. In any case, SAH-related mortality is certainly not related to hemorrhage-induced early global cerebral ischemia since the duration of global cerebral ischemia typically observed after SAH, that is, 2-3 minutes, does not cause any mortality in comparable experimental models of global cerebral ischemia; in these models, more than 8 minutes of three/four vessel occlusion are necessary to produce at least some mortality [47, 48]. In addition, mortality after experimental global ischemia typically occurs between day 3 and day 5 after the insult and not within the first three days like after experimental SAH. Accordingly, the mortality observed after SAH in rodents does not seem to be caused by global ischemia, but rather by later changes associated with blood-brain barrier opening, microcirculatory failure, and focal cerebral ischemia.

## 3. Pathophysiological Findings following Experimental Global Cerebral Ischemia

Cardiac arrest results in an immediate drop in systemic blood pressure and a subsequent cessation of cerebral blood flow resulting in global cerebral ischemia. The lack of cerebral blood flow results in anaerobic metabolism leading to tissue acidosis, anoxic depolarization of neuronal cells with release of glutamate and other neurotransmitters into the extracellular space, and in immediate swelling of glial cells. As a consequence, extracellular glutamate concentrations increase by several folds and cytotoxic edema develops [41, 49]. If the restoration of cardiac function occurs before the respiratory centers of the brain stem are permanently damaged, survival is possible [50]. Usually reperfusion of the brain is followed by a hyperemic response [51], and large as well as small cerebral vessels are fully perfused within a few minutes [52]. Despite sufficient cerebral blood flow, usually neuronal cell death occurs with a delay of 3–5 days in the hippocampus [53] and in selective cortical areas resulting mainly in memory and executive function deficits [54–56]. These events are found in a very similar manner in experimental animals as well as in patients who suffered a cardiac arrest.

## 4. Similarities between Experimental Global Cerebral Ischemia and SAH

When comparing the pathophysiology observed after global cerebral ischemia and SAH, it becomes quite obvious that the early phase of SAH shows some phenomena which are very similar to those observed after global cerebral ischemia. In both conditions, cerebral blood flow may come to a complete stop or is at least reduced below the ischemic threshold of 20% of physiological cerebral blood flow [33, 35], and extracellular glutamate concentrations are increased significantly for at least 30–60 min [24, 33, 49]. These changes in blood flow trigger an acute activation of cerebrovascular endothelial cells and cause a delayed but transient interaction of inflammatory cells and platelets with cerebral vessels for only a few hours [52, 57]. The glutamate releases triggered by global cerebral ischemia and SAH result in excitotoxicity and delayed neuronal cell death selectively in the hippocampus and in subsequent memory and executive function deficits [25, 49].

Similarities between global cerebral ischemia and SAH are also found during the reperfusion phase which occurs after restoration of cerebral blood flow. Provided animals have a sufficiently high blood pressure [38, 51], reperfusion occurs within a few minutes and results in full restoration of flow in large and small cerebral vessels as well as on the level of the microcirculation [25, 48, 52, 57]. When blood pressure is not sufficiently high, both conditions result in slow or lacking reperfusion which results in low survival rates and exacerbation of delayed brain injury [33, 38, 45, 51].

## 5. Dissimilarities between Experimental Global Cerebral Ischemia and SAH

As soon as the acute phase of global cerebral ischemia and SAH is over, the pathophysiology of both conditions starts to show a growing number of dissimilarities. The main reason for this observation is the persisting triggering of further pathophysiological processes by the presence of blood in the subarachnoid space following SAH while in global cerebral ischemia the pathophysiology certainly proceeds, but no further pathophysiological events are additionally initiated [41, 51]. One important feature of SAH not found in global cerebral ischemia is the acute constriction of large intracranial vessels [19, 33] and the subacute occurrence of cerebral microvasospasm and microthrombosis [21, 58] in areas of subarachnoid blood deposition. These vascular changes may well prolong and/or exacerbate the perfusion deficits acutely caused by global ischemia and result in delayed focal cerebral ischemia and the formation of delayed brain edema as also observed in SAH patients [15, 22, 59]. 

## 6. Summary and Conclusion

The current literature as well as our own results suggests that the early pathophysiology of SAH consists of two phases: one related to the brief global ischemia caused by the initial bleeding and one linked to the vascular damage caused by the blood ensheathing the brain supplying arteries in the subarachnoid space. This concept is further supported by the fact that microvessels in the subarachnoid space adjacent to the cerebral cortex are functionally impaired, that is, do not react to CO_2_ (unpublished data), show microvasospasms [19, 21], are prone to develop microthrombosis [21, 58], and show progressive opening of the blood-brain barrier [42]. Accordingly, events induced by global cerebral ischemia are well present after SAH and play an important pathophysiological role but represent only one out of many important components of the complex pathophysiology of SAH.
